# Heat Adaptive Capacity: What Causes the Differences Between Residents of Xiamen Island and Other Areas?

**DOI:** 10.3389/fpubh.2022.799365

**Published:** 2022-02-21

**Authors:** Chaowei Wu, Wei Shui, Haifeng Yang, Meiqi Ma, Sufeng Zhu, Yuanmeng Liu, Hui Li, Furong Wu, Kexin Wu, Xiang Sun

**Affiliations:** ^1^Department of Geography and Planning, College of Environment and Safety Engineering, Fuzhou University, Fuzhou, China; ^2^Center for Urban Security Development Research, College of Architecture and City Planning, Nanjing University, Nanjing, China; ^3^Chinese Research Academy of Environmental Science, Beijing, China

**Keywords:** climate change, extreme heat events, heat vulnerability, adaptive capacity, human-environment system, Xiamen Island, China

## Abstract

Extreme heat events caused by climate change have serious adverse effects on residents' health in many coastal metropolises in southeast China. Adaptive capacity (AC) is crucial to reduce heat vulnerability in the human-environment system. However, it is unclear whether changes in individual characteristics and socioeconomic conditions likely amplify or attenuate the impacts of residents' heat adaptive capacity (HAC) changes. Moreover, which public policies can be implemented by the authorities to improve the HAC of vulnerable groups remains unknown. We conducted a questionnaire survey of 630 residents of Xiamen, a typical coastal metropolis, in 2018. The effects of individual and household characteristics, and government actions on the residents' HAC were examined by using ordinal logistic regression analysis. Results show that the majority (48.10%) of Xiamen residents had a “medium” HAC level, followed by a “high” level (37.14%). On Xiamen Island, residents who settled locally for one–three years and spent less than one hour outdoors might report weaker HAC, and their HAC would not improve with increased air conditioning units in household. In other areas of Xiamen, residents with more rooms in their households, no educational experience, and building areas <50 m^2^ might report better HAC. Further, vulnerable groups, such as local residents and outdoor workers on Xiamen Island, people lacking educational experience and renters in other areas of Xiamen, showed better AC to hot weather than those in previous studies. Low-income groups should be given more attention by local governments and community groups as monthly household income played a positive role in improving Xiamen residents' HAC. Rational green spaces planning and cooling services, such as street sprinkling operations, provided by municipal departments can effectively bring benefits to Xiamen residents. Identification of basic conditions of AC has significant implications for practical promoting targeted measures or policies to reduce health damages and livelihood losses of urban residents during extreme heat events.

## Introduction

Extreme heat events around the world are occurring more frequently due to climate change caused by human activities (1). These events have gradually evolved into severe meteorological disasters (2) and have adversely affected the development of human society and health (3, 4). In the past few decades, continuous urbanization has increased the intensity and duration of hot days in coastal metropolitan areas (5, 6). Due to the accelerated concentration of population and capital, extreme heat events are becoming more frequent in developed areas along the southeast coast of China (7–9), such as Xiamen.

**Figure 1:**
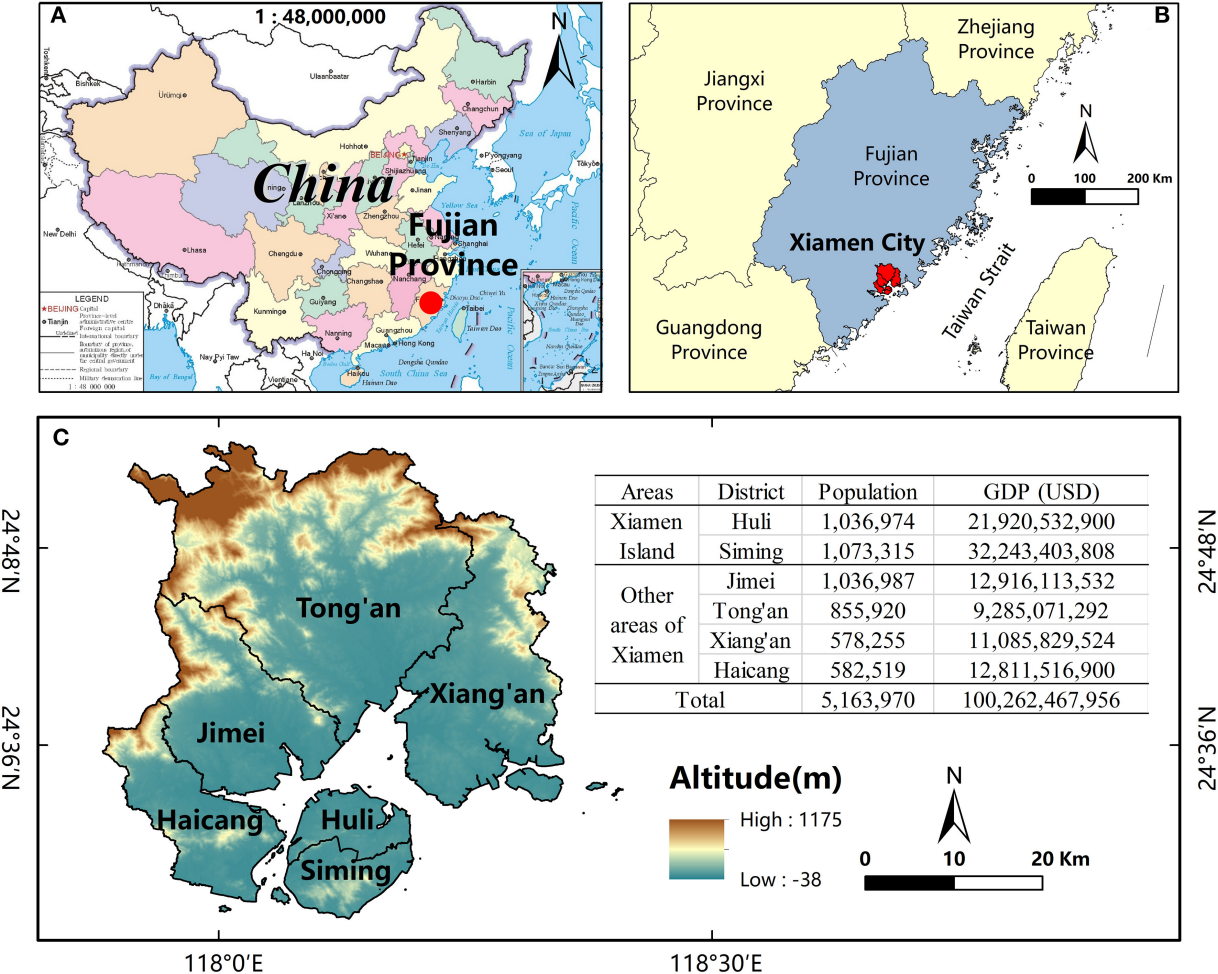
The location of Xiamen City. **(A)** Fujian Province in China; **(B)** Xiamen City in Fujian Province; **(C)** the six districts and elevation map of Xiamen City. Background map source: National Geomatics Center of China. Population data source: the 7th National Census (2020). GDP data source: 2021 Yearbook of Xiamen Special Economic Zone.

Xiamen, an important central city and tourist location, has nearly 4.11 million inhabitants. It has a total area of 1700.61 km^2^, of which the land area of Xiamen Island is 157.98 km^2^ (including Gulangyu), and the sea area is ~390 km^2^. There are six districts in the Xiamen metropolitan area, among which Siming and Huli are located on Xiamen Island, while Haicang, Jimei, Tong'an, and Xiang'an are located in other areas. Since the early 1980s, temperatures in Xiamen have continued to rise, and heat events have occurred more frequently. According to meteorological data from the Fujian Meteorological Bureau, there were 24 heat wave events from 1980 to 2014, with an average temperature rise of 0.43°C per decade and extreme temperatures of up to 39.2°C. Under the influence of buildings and background winds (10), the intensity of Xiamen's urban heat island may further increase, leading to an expansion of the adverse effects of extreme heat events. Despite Xiamen Island having a large population, favorable economic and social conditions, and an attractive natural environment (Figure 1), there has been little verification of whether the residents of Xiamen Island are better able to cope with the health threats of hot weather.

Extreme heat events have threatened the health and livelihood of coastal metropolitan residents (11–13). It has been proven that there is a significant correlation between extreme heat and people's subjective health status. The incidence of cardiovascular, respiratory, and digestive tract diseases caused by scorching weather is on the rise (14–16). However, it is still unclear which groups in coastal metropolitan areas are more sensitive or vulnerable to hot weather, and there is an urgent need to know what measures can people take to cope with potential heat health threats and what targeted policies, such as welfare and public investment, can be developed by community groups and local governments to reduce the loss of life, health, and property during extreme heat events. In the face of these problems, we should treat human society and the natural environment as a unified unit (17). National Research Council of the National Academy of Sciences (18) believes that understanding why some people or regions are better able to cope with the negative impacts of climate change should focus on the geographical differences in exposure, vulnerability, and AC from the perspective of vulnerability, and adaptive capacity of the human-environmental system under external stress (extreme heat).

IPCC's report (19) defines AC as “the ability of systems, institutions, humans, and other organisms to adjust to potential damage, to take advantage of opportunities, or to respond to consequences.” The report affirms the importance of AC in assessing vulnerability to climate change. As a crucial component of the vulnerability of the human-environment system, AC can mobilize scarce resources to cope with expectations or current pressures, thus affecting the ultimate potential of achieving sustainable adaptation (20). Therefore, knowing and understanding HAC has become critical for the development of health policies and adaptation actions to enhance the ability of residents to cope with heat stress. HAC studies can also provide information for understanding the primary conditions of adaptation to extreme heat to support the governments, stakeholders, and residents in governance and decision-making. Recently, scholars have attempted to incorporate AC into the conceptual framework for constructing urban heat vulnerability evaluation models in the coupled study of urban heat hazards and human health. These studies used socio-demographic factors (e.g., age, gender), economic status, and incidence of chronic disease as indicators for assessing AC (21–26).

Adaptation is a complex problem (27). AC is measured differently at various temporal and spatial scales, in different cultural contexts, and with different social objectives (22, 28–31). The AC of an individual, city, or community is often affected by a series of decisive factors (29, 32). Therefore, it is imperative to determine how AC is built, what constitutes it, and what hinders or limits it (28). A research report (33), co-authored by the European Topic Centre on Climate Change Impacts, Vulnerability and Adaptation and the European Topic Centre on Spatial Information and Analysis, made a proposal to focus on AC from three aspects: “Ability,” “Action,” and “Awareness.” However, the quantitative measurement and spatial characterization of residents' HAC remain a challenging subject, and few studies have been devoted to exploring the means for residents to enhance their AC in the face of pressure in hot weather. From the perspective of the human-environment system, it is necessary to explore residents' HAC in terms of individual characteristics, household characteristics, government actions (34, 35), which allows us to get insight into the mechanisms by which people and the environment work together to cope with external interference (extreme heat) and to explain the composition of factors influencing HAC. In addition, current research on urban heat vulnerability or AC may not be interpreted at the level of individuals and their perceptions, which may have implications for the effective implementation of integrated person-centered health policies. It is increasingly recognized that the development of appropriate adaptation strategies requires a deeper understanding of the impacts of climate change on human beings (13, 36). Although it is relatively rare to collect information about residents' HAC through interviews or questionnaires, these methods are very effective for making observations about exposure, sensitivity, and AC at multiple scales (18, 37–39).

In this study, a questionnaire survey was conducted among the residents of Xiamen to: (1) explore their HAC and identify its significant influencing factors in terms of individual characteristics, household characteristics, government actions; (2) highlight the differences in their HAC and the influencing factors between Xiamen Island and other areas of Xiamen. Identifying these conditions can provide a scientific reference for policymakers to develop implementable health policies to improve the HAC of residents in metropolitan areas and reduce the health damage caused by extreme heat.

## Materials and Methods

### Selection of Characteristics Associated With Residents' HAC

Residents' HAC should be examined on both a small-scale (individuals and households) and a large-scale (local governments and community groups). To investigate HAC on a smaller scale, individual HAC scales should include personal cognition, attitude, and the methods by which people protected themselves from heat stress (33, 40). HAC, on the individual and household scale, can be measured in terms of age, education level, health status, household economic status, housing conditions, information access, and other factors (22, 24, 41–43). To examine HAC on a larger scale, the impact of cooling programs and services provided by the community and municipality on the behaviors and ability of individuals to withstand heat waves should be investigated, since the residents directly experience and benefit from these services and facilities. HAC, on a larger scale, is influenced by the construction of urban cooling facilities, medical support configuration, heat forecasts, early warning information pushing, municipal cooling operations, and landscape configuration, among others (22, 24, 41, 44). This can serve as a reference for recommendations on the facilities and services that local governments should provide to cope with urban heat waves. Based on the perspective of the human-environment system, this approach is conducive to a deeper understanding of the challenges, needs, and practices of the city in the face of extreme heat events.

Combining the literature point of view and the consultation results of experts in related fields, we list the characteristics associated with residents' HAC that were used in our questionnaire and analysis (Table 1).

**Table 1:** Characteristics associated with residents' HAC.

**Scales**	**Indicators**	**Characteristics**
Individuals	Personal characteristics	Gender (45, 46)
		Age (21, 47)
		Body Mass Index (24)
		Education level (21–23)
		Health status (22)
		Hours spent outdoors per day (48)
	Awareness and action to prevent heat waves	Obtain hot weather information initiatively (40)
		Go out for cooling centers initiatively (40)
Households	Household characteristics	Number of family members (49)
		Monthly household income (22, 47)
		Building area (50)
		Number of air conditioning units in household (43, 51)
		Number of fans in household
		Years of local residence
		Number of rooms in household
Local governments and community groups	Convenience of accessing various facilities or spaces	Access to cooling facilities (21, 41)
		Access to medical support facilities (45)
		Access to public transportation facilities
		Access to river-waterfront spaces (21)
		Access to green spaces (52, 53)
	Frequency of various services provided by community and municipality	Frequency of releasing hot weather information (40) Frequency of street sprinkling operations by municipal departments

### Data Collection

Data collection was divided into two steps: questionnaire design and formal implementation. In the first stage, the questionnaire was designed objectively based on the 22 characteristics listed in Table 1 (the questionnaire can be obtained in Supplementary Data Sheet 1). Residents living in Xiamen City were the target population for the survey. A five-point Likert scale was used to measure subjective questions, which facilitated the subsequent quantitative analysis. At the end of the questionnaire, participants were asked to rate their own HAC based on their responses to the previous questions. A semantic difference scale was used to classify the rating scale into five levels: lowest, low, medium, high, and highest. The scale and questions were evaluated by several experts before the questionnaire was completed. A pre-survey was conducted, and the questionnaire was further optimized based on the feedback. Finally, the survey was conducted in Xiamen, China from August 7 to August 14, 2018, employing the formal questionnaire.

Survey sites were determined based on spatial and random sampling. A total of 57 locations were selected in Xiamen Island and other areas of Xiamen, mainly in parks, shopping complexes, universities, and residential areas. The participants completed the questionnaire independently. For those with limited capacity to read and write, the investigator dictated the questions and filled in their answers. To ensure the authenticity and validity of the information, the questionnaires were filled in for 10–30 min. A total of 691 questionnaires were distributed, and 630 valid questionnaires were returned, with a valid return rate of 91.17%.

### Statistical Method

For data analysis, IBM SPSS 21.0 (International Business Machines Corporation, Armonk, NY, USA) was used. To identify the differences between Xiamen Island and other areas of Xiamen in terms of individual characteristics, household characteristics, and government actions, a descriptive analysis was conducted, followed by ANOVA and Chi-square test. Finally, an ordinal logistic regression model was used to identify the factors that significantly influenced residents' HAC. The ordinal logistic regression model was established according to the following steps: ① suspicious variables affecting residents' HAC were analyzed using a univariate ordinal logistic regression model, and the statistically significant variables were selected (significance level was set at *p* < 0.15); ② screened variables in Step 1 were diagnosed by multicollinearity, and variables with a variance inflation factor >2 were eliminated; ③ the remaining variables were analyzed using an ordinal logistic regression model (significance level was set at *p* < 0.05).

### Model Specification

This study used a logistic regression model to further identify the factors influencing residents' HAC. The independent variables of the model are the characteristics selected in Table 1, which have been conceptualized as questions in the questionnaire. The options for the questions were coded as numbers before the analysis for easy input into the model. Residents' HAC was chosen as the dependent variable. Since the dependent variable was ordinal, the ordinal logistic regression model was chosen for this study.

Suppose the dependent variable Y has k levels, and the probability of each level was explained as π_1_, π_2_, π_3_,…, π_*k*_, then π_1_+π_2_+π_3_+…+π_*k*_ = 1. The effect of *P* different factors (the explanatory variables were denoted as *x*) on the probability of each category of the explanatory variables can be analyzed as a *k*−1 model (54), constructed as follows:


(1) $$\begin{array}{r} -\ln\left[-ln\left(\pi_{1}\right)\right]=\alpha_{1}+\beta_{1}x_{1}+\ldots +\beta_{p}x_{p} \end{array}$$

(2) $$\begin{array}{r} -\ln\left[-ln\left(\pi_{1}+\pi_{2}\right)\right]=\alpha_{2}+\beta_{1}x_{1}+\ldots +\beta_{p}x_{p} \end{array}$$

(3) $$\begin{array}{r} -\ln\left\{-\ln\left[\pi_{1}+\pi_{2}+\ldots +\pi_{k-1}\right]\right\}=\\ \;\alpha_{k-1}+\beta_{1}x_{1}+\ldots +\beta_{p}x_{p} \end{array}$$

where α is the threshold (constant term), and β is the position parameter (regression coefficient).

## Results

Supplementary Tables 2, 3 show that the proportion of male participants is greater than females (55.40% males and 44.60% females), comparable to the gender distribution of the resident population in Xiamen in the 7th National Census in 2020 (52.68% males and 47.32% females). The results of the Chi-square test show that there were no statistically significant differences in the characteristics associated with participants' HAC, except for the number of fans in household, regardless of whether the participants lived on Xiamen Island or other areas of Xiamen. Overall, 92.06% of participants reported their HAC as “medium” or above, of which almost half (48.10%) described their HAC as “medium.” (Supplementary Tables 2, 3 can be obtained in Supplementary Data Sheet 2).

Table 2 presents the three ordinal logistic regression models constructed in this study. The models are statistically significant, the degree of fit is good, and the hypothesis of “comparative advantage” of the model is established. The parallel lines test shows that the individual regression equations created by the model are parallel to each other. Therefore, the three models were suitable for studying the factors influencing residents' HAC.

**Table 2:** Factors significantly influencing residents' HAC based on results of ordinal logistic regression.

**Factors**	**Model I**			**Model II**			**Model III**		
	**(All areas of Xiamen)**			**(Xiamen Island)**			**(Other areas of Xiamen)**		
	**Estimate**	**95*****%*** **CI**		**Estimate**	**95*****%*** **CI**		**Estimate**	**95*****%*** **CI**	
		**Lower**	**Upper**		**Lower**	**Upper**		**Lower**	**Upper**
Number of air conditioning units in household	-	-	-	–0.092^*^	–0.179	–0.006	-	-	-
Number of rooms in household	0.095^*^	0.009	0.182	-	-	-	0.195^**^	0.054	0.337
[Education level = 1]	4.297^***^	2.084	6.511	-	-	-	3.607^*^	0.836	6.379
[Education level = 2]	0.631	−0.535	1.798	-	-	-	−0.982	−2.943	0.979
[Education level = 3]	0.411	−0.110	0.932	-	-	-	0.249	−0.499	0.997
[Education level = 4]	0.231	−0.176	0.638	-	-	-	0.486	−0.126	1.098
[Education level = 5]	0^a^	-	-	-	-	-	0^a^	-	-
[Hours spent outdoors per day = 1]	–1.051^**^	–1.772	-0.330	–1.283^*^	–2.416	–0.150	-	-	-
[Hours spent outdoors per day = 2]	−0.312	−0.936	0.312	−0.365	−1.365	0.635	-	-	-
[Hours spent outdoors per day = 3]	−0.602	−1.258	0.054	−0.745	−1.811	0.322	-	-	-
[Hours spent outdoors per day = 4]	−0.363	−1.138	0.412	0.503	−0.854	1.860	-	-	-
[Hours spent outdoors per day = 5]	0^a^	-	-	0^a^	-	-	-	-	-
[Building area = 1]	-	-	-	-	-	-	1.808^*^	0.134	3.481
[Building area = 2]	-	-	-	-	-	-	0.663	−0.875	2.201
[Building area = 3]	-	-	-	-	-	-	0.488	−0.973	1.949
[Building area = 4]	-	-	-	-	-	-	0.456	−1.199	2.111
[Building area = 5]	-	-	-	-	-	-	0^a^	-	-
[Years of local residence = 1]	-	-	-	0.144	−0.821	1.109	-	-	-
[Years of local residence = 2]	-	-	-	–0.880^*^	–1.712	–0.048	-	-	-
[Years of local residence = 3]	-	-	-	0.047	−0.783	0.877	-	-	-
[Years of local residence = 4]	-	-	-	−0.303	−1.045	0.438	-	-	-
[Years of local residence = 5]	-	-	-	0^a^	-	-	-	-	-
[Monthly household income = 1]	–1.486^***^	–2.285	–0.687	−1.117	−2.296	0.063	–1.989^**^	–3.231	–0.747
[Monthly household income = 2]	–0.942^**^	–1.621	–0.264	–1.446^**^	–2.440	–0.453	−0.936	−1.931	0.059
[Monthly household income = 3]	–0.842^**^	–1.401	–0.283	−0.576	−1.430	0.278	–1.339^**^	–2.167	–0.510
[Monthly household income = 4]	−0.441	−0.997	0.115	−0.640	−1.490	0.211	−0.549	−1.395	0.297
[Monthly household income = 5]	0^a^	-	-	0^a^	-	-	0^a^	-	-
[Access to green spaces = 1]	0.335	−0.922	1.591	–2.795^*^	–5.065	–0.525	0.745	−0.761	2.252
[Access to green spaces = 2]	–1.074^**^	–1.853	–0.296	–1.754^**^	–3.015	–0.493	–1.274^*^	–2.428	–0.120
[Access to green spaces = 3]	–0.878^*^	–1.596	–0.161	–1.873^**^	–3.072	–0.674	−0.462	−1.420	0.495
[Access to green spaces = 4]	−0.507	−1.090	0.076	–1.390^**^	–2.429	–0.351	−0.108	−0.830	0.614
[Access to green spaces = 5]	0^a^	-	-	0^a^	-	-	0^a^	-	-
[Frequency of street sprinkling = 1]	–0.970^*^	–1.833	–0.108	-	-	-	-	-	-
[Frequency of street sprinkling = 2]	–0.824^*^	–1.520	–0.127	-	-	-	-	-	-
[Frequency of street sprinkling = 3]	−0.534	−1.199	0.132	-	-	-	-	-	-
[Frequency of street sprinkling = 4]	−0.449	−1.093	0.196	-	-	-	-	-	-
[Frequency of street sprinkling = 5]	0^a^	-	-	-	-	-	-	-	-
Model Fitting Information:	*χ^2^* = 131.870, *p* < 0.001			χ^2^ = 88.304, *p* < 0.001			*χ^2^* = 83.748, *p* < 0.001		
Goodness of Fit:	Pearson *χ^2^* = 2628.604, *p* < 0.01			Pearson *χ^2^* = 1475.595, *p* < 0.001			Pearson *χ^2^* = 1258.823, *p < * 0.01		
Cox and Snell	0.192			0.282			0.245		
Test of Parallel Lines:	–2 Log Likelihood: 1097.708, *χ^2^* = 166.293, *p* = 0.457			–2 Log Likelihood: 419.653, *χ^2^* = 86.025, *p* = 0.999			–2 Log Likelihood: 459.365, *χ^2^* = 147.220, *p* = 0.223		

*Link function: Logit*.

a *This parameter is redundant; thus, it is set to zero*.

* *Significant at the 0.05 level*.

** *Significant at the 0.01 level*.

*** *Significant at the 0.001 level*.

### All Areas of Xiamen

Table 2 shows that among individuals' characteristics, education level and hours spent outdoors per day had significant effects on participants' HAC. Participants with no educational background were likely to have higher HAC than those with education level at university and above. Participants who spent less than one hour outdoors showed a significant disadvantage in HAC compared to those who spent more than eight hours outdoors.

From the household aspects, the number of rooms in household and monthly household income had a positive effect on participants' HAC. As the number of rooms in household increased, participants' HAC would significantly improve. Participants with a monthly household income of more than RMB 20,000 were likely to report better HAC than those with a monthly household income of < RMB 2,000, RMB 2,000–5,000, and RMB 5,000–10,000.

Neither variable had a significant effect on participants' HAC in terms of their awareness and action to prevent heat-stroke. However, the convenience of accessing green spaces significantly affected participants' HAC, as demonstrated by the participants who found it “hard” and “general” to access green spaces were likely to report lower HAC compared to participants who found it “very easy” to access green spaces. When participants perceived that the municipalities “never” and “seldom” sprinkled water on the streets, they were likely to report lower HAC than those who perceived that the municipalities “always” sprinkled water on the streets.

### Xiamen Island and Other Areas of Xiamen

Table 2 shows that the factors significantly influencing the participants' HAC on Xiamen Island and those in other areas of Xiamen were different.

Participants from Xiamen Island reported their HAC that was not influenced by their education background, but participants from other areas of Xiamen with no educational background reported significantly better HAC than those with university and higher education level. For participants living on Xiamen Island, if they spent less than one an hour outdoors per day, their HAC was more likely to be weaker than those who spent more than eight hours outdoors. In contrast, those living in other areas of Xiamen were not affected by the hours spent outdoors per day.

In terms of household characteristics, participants' HAC was influenced by monthly household income, regardless of whether they lived on Xiamen Island or in other areas. Participants living on Xiamen Island with a monthly household income of more than RMB 20,000 were likely to report better HAC than those with RMB 2,000–5,000. However, in other areas of Xiamen, participants with a monthly household income of more than RMB 20,000 were likely to report better HAC than those with < RMB 2,000 and those with RMB 5,000–10,000.

Years of local residence and number of air conditioners units in household had significant effects on the participants' HAC on Xiamen Island, indicating that participants who had lived locally for only one–three years might report weaker HAC than those who had lived locally for more than 10 years, and that participants' HAC might decrease as the number of air conditioners units in household increased. However, the above two factors had no impact on participants living in other areas of Xiamen, whose HAC was chiefly influenced by building area and number of rooms in household. Participants with <50 m^2^ of building area were likely to report higher HAC than those with more than 200 m^2^. On the other hand, participants with more rooms in their households might report stronger HAC.

The convenience of accessing green spaces had significant effect on participants' HAC regardless of whether they lived on Xiamen Island or other areas of Xiamen. On Xiamen Island, participants who found it “very difficult,” “difficult,” “fair,” and “easy” to access green spaces were likely to report weaker HAC compared to those with green spaces close at hand, while for participants living in other areas of Xiamen, only those who found it “difficult” to access green spaces might report weaker HAC.

## Discussion

This survey reflected the potential need of Xiamen residents to withstand hot weather. Unlike previous studies that used macroeconomic statistics (22, 41, 55), this study used semi-structured interviews to obtain a large sample of data, which provided a more comprehensive picture of residents' HAC. In the past, the Siming and Huli (Xiamen Island) had advantages over the four districts (other areas of Xiamen) in many aspects such as population, education, capital, healthcare, natural environment; the region's ability to cope with high temperatures could have been enhanced employing these. The Xiamen government had been actively implementing an integration strategy to promote the joint development of Xiamen Island and other areas of Xiamen in recent years. The local governments took equity and fairness into account in the integrated development of metropolitan areas. Supplementary Table 3 shows that the participants' HAC did not show statistically significant differences between Xiamen Island and other areas of Xiamen, which also indicated the achievements of the Xiamen government's efforts in the fields of employment, education, and urban infrastructure construction.

In this study, factors such as gender, age, BMI, health status, and the number of family members had no significant effects on residents' HAC. This is inconsistent with findings suggesting that the elderly and people with poor health status were heat sensitive and faced a higher health risk (22, 56, 57). It is to be noted that the proportion of children and elderly population in the study was small, and which might lead to a bias of the level of residents' HAC. However, this finding is similar to research conducted in Oakland, a coastal metropolis adjacent to San Francisco Bay. While a high percentage of low-income individuals lived in the urban center of Oakland, they did not demonstrate significant heat vulnerability and poor AC due to the lack of green spaces, old age, or health issues (22). Xiamen is a coastal metropolis with a large local population of older adults (according to the data of the 7th National Census, the population of Xiamen aged 60 and above was 493,579, accounting for 9.56%; an increase of 2.63% from the 6th National Census). Recently, Xiamen had established an “old-age service system” that is “home-based, community-supported, institutionally supplemented, and medical care-combined” covering both urban and rural areas (58). Xiamen's well-developed medical infrastructure and “old-age service system” can help treat the elderly who have suffered from the effects of heat waves.

Our results indicated that participants who lacked educational experience might report stronger HAC. However, our study might not provide a good representation of this group since only four such participants were surveyed. People with only high school education or less are associated with higher rates of heat-related deaths in previous studies (22, 59). Further research may be needed to investigate whether groups lacking educational experience are vulnerable and susceptible to heat in a coastal Chinese metropolis such as Xiamen. This finding also indicated no statistical difference in the ability of the participants with higher education levels to cope with hot weather. In Supplementary Table 3, the education level of participants was mainly concentrated at the university level and above. It is undeniable that residents with higher education levels have more experience and knowledge dealing with the health threats of hot weather.

We found that participants who spent less than an hour outdoors reported significantly weaker HAC than those who spent more than 8 h outdoors, especially in participants from Xiamen Island. This finding, to the best of our knowledge, is novel. During the study, we also found that many people who worked long hours outdoors, such as food delivery workers, leaflet distributors, marketing staff, and sanitation workers, indicated strong AC to hot weather. While they believed that the heat makes their work more difficult and burdensome, they were not afraid. Another interesting finding in our results is that the more air conditioning units the participants had in their households on Xiamen Island, the lower their HAC. Cheung and Jim (43) found that air conditioning units in poorly ventilated houses led to the deterioration of indoor air quality, and pressure in hot weather was also observed. Poor ventilation was associated with sick building syndrome (SBS) (51, 60). Therefore, we recommend that residents living on Xiamen Island do not stay indoors for a long time, and opening windows to get fresh air is essential.

Monthly household income had been affirmed to reduce heat vulnerability for residents in several studies (22, 24, 25, 41), this is consistent with the results of our study. Low-income individuals in Seoul had a higher mortality rate during hot weather (61). Low-income families also tended to have higher heat vulnerability (25) and lack air-conditioning equipment (37). To be sure, income, as found in previous studies, also plays a role in reducing heat risk for individuals and households in coastal metropolitan areas.

Our survey results showed that most participants (43.46%) from Xiamen Island had resided there locally for more than 10 years. They reported significantly higher HAC than those who had only lived there for one–three years. However, notably, the effect of years of local residence was not significant for participants from other areas of Xiamen. Compared to migrant workers and graduates who have lived locally for a shorter period, those who settled in Xiamen Island for a more extended period were more aware of the heat wave patterns occurring in the area. Since they know how to access cooling and medical resources faster, they may be better equipped to cope with the heat. This reflects the importance of personal experiences in withstanding natural disasters (62).

Our findings showed that more rooms in household might have stronger HAC, as reported by participants in the other areas of Xiamen. The number of rooms and building areas indirectly reflects the living conditions and economic status of the residents. Typically, the larger the living area, the better the ventilation and space for activities. Lim and Skidmore (25) found that people living in mobile or rented homes were more vulnerable to extreme heat. However, participants living in houses with a building area of <50 m^2^ in the other areas of Xiamen reported stronger HAC. This may reflect a unique group; renters, who share a whole house, to save money despite the small sizes of the rooms. It also indicates that these groups in Xiamen may not rely on the cooling environment provided by their housing. The methods and resources renters utilize to cope with heat are worth exploring further.

In our survey, participants who rarely obtained information about hot weather (29.74 and 31.48% for Xiamen Island and other areas of Xiamen, respectively) and who were less likely to go out to find cooling centers (33.01 and 35.80% for Xiamen Island and other areas of Xiamen, respectively) accounted for a large proportion of the participants. The participants did not seem interested in obtaining information about the hot weather and cooling centers. Considering the pleasant climate of coastal metropolitan areas, residents may not have worried about the effects of high temperatures. In Xiamen, the average number of air conditioning units reported by participants was 2.43. Since they had one fan in their households at least, it was considered adequate cooling equipment, and there was no need for them to go out and find a public cooling center initiatively.

Various facilities and cooling services, especially green spaces and sprinkling water on the streets, provided by the local governments and community groups, had been confirmed in this study to have a positive impact on residents' HAC. Green spaces have been shown to alleviate the urban heat island effect (52). They provided health benefits for residents who can enjoy staying under the shade of trees (53), which could help improve personal HAC. For cities, sprinkling water on the streets is an effective measure for mitigating a surface urban heat island (63). Which can also contribute to a cooler and healthier living environment for urban residents.

### Limitations

One major drawback of this study is that it was only conducted in one metropolis along the southeastern coast of China. The sample size of 630 participants is not representative of the entire population of Xiamen, nor is it representative of coastal metropolises like Xiamen. Although this study involved the specific characteristics of individuals coping with heat risk in material and economic aspects, it did not explore the impact of environmental differences in nature and culture on residents' HAC. Furthermore, we could not completely rule out subjectivity in the questionnaire survey. Finally, not all the results can be directly compared with those of other studies, and further comparative studies are needed to extend the approach of this study to other coastal metropolises.

## Conclusion

In the context of global warming, metropolises are increasingly becoming threatened by extreme heat such that those lacking the measures and capacity to withstand heat-related disasters may experience severe losses. It is essential to focus on the regional prerequisites of Xiamen compared with other countries and climate zones, and gain insight into residents' HAC at multiple scales for detailed, targeted strategies against extreme heat. For this, a questionnaire survey was conducted among residents of Xiamen to gather information about their HAC. The findings and methods employed in this study can be easily extended to other similar coastal metropolitan areas. This investigation is vital for urban residents and local governments because extreme heat events are directly related to human health and community well-being. For cities, authorities urgently need to develop people-centered preparedness plans and disposal measures to reduce heat disaster losses and achieve sustainable urban development for the decades to come.

There is a spatial heterogeneity in the factors influencing residents' HAC, and the influencing mechanisms of individual characteristics and socioeconomic conditions also vary across different areas of Xiamen city. In our study, groups sensitive to heat in previous studies, including local residents and outdoor workers on Xiamen Island and people with lower education levels and renters in other areas of Xiamen, did not show low HAC levels. Further research should explore why these groups may be well-equipped to cope with hot weather.

Our findings confirmed the role of monthly household income, the convenience of accessing green spaces, and city cooling services in improving residents' HAC and living environments. Local governments and community groups should implement targeted plans to prevent potential health injuries to residents with lower household income levels during extreme heat events. Authorities in Xiamen should create plans for urban green spaces and provide cooling services, like sprinkling water on the streets during hot days, which would mitigate the damage caused by extreme heat events and improve residents' HAC.

## Data Availability Statement

The original contributions presented in the study are included in the article/Supplementary Material, further inquiries can be directed to the corresponding author.

## Ethics Statement

Approval of all ethical and experimental procedures and protocols was granted by the Ethics Committee of Fuzhou University. Written informed consent to participate in this study was provided by the participants' legal guardian/next of kin.

## Author Contributions

WS: conceptualization and research design. CW: software and results analysis. CW and HY: writing—original draft preparation. CW, HY, MM, SZ, YL, HL, FW, KW, and XS: review and editing. All authors have read and agreed to the published version of the manuscript.

## Funding

This research was funded by National Key Research and Development Plan Program of China (2016YFC0502905).

## Conflict of Interest

The authors declare that the research was conducted in the absence of any commercial or financial relationships that could be construed as a potential conflict of interest.

## Publisher's Note

All claims expressed in this article are solely those of the authors and do not necessarily represent those of their affiliated organizations, or those of the publisher, the editors and the reviewers. Any product that may be evaluated in this article, or claim that may be made by its manufacturer, is not guaranteed or endorsed by the publisher.

## Abbreviations

HAC: heat adaptive capacity
AC: adaptive capacity.
