# Supplementary material for: Heat Adaptive Capacity: What Causes the Differences Between Residents of Xiamen Island and Other Areas?
Source: Front Public Health. 2022 Feb 21;10:799365. doi: 10.3389/fpubh.2022.799365 (PMC8899036; doi:10.3389/fpubh.2022.799365)
Supplement: Supplementary file 1 [file Data_Sheet_1.docx]

Supplementary Material S1

For reproduction purposes, and for a better relation to the survey elements, the statements used in the questionnaires have been translated into English and can be found in this document.

**Questionnaire of Xiamen Residents’ Adaptive Capacity to Hot Weather in the Summer**

Dear friends:

Hello everyone! The purpose of this questionnaire is to provide suggestions to improve Xiamen residents’ ability to cope with urban heat by investigating their personal and household characteristics, as well as their perceptions of the cooling facilities and services provided by local governments and community groups. Please fill in the blanks and check the selected answers according to your information and judgment. Thank you for your support.

1. **Gender: ; Age: ; Height: ; Weight: .**
2. **Which Districts do you live in?**

A: Siming B: Huli C: Jimei D: Haicang E: Tong’an F: Xiang’an

1. **Number of members in your family: ; Number of rooms in your household： ; Number of air conditioning units in your household： ; Number of fans in your household： .**
2. **How long have you settled locally?**

A: less than a year B: 1–3 years C: 3–5 years D: 5–10 years E: more than 10 years

1. **What is your household’s monthly income?**

A: less than 2000 RMB B: 2000–5000 RMB

C: 5000–10000 RMB D: 10000–20000 RMB E: more than 20000 RMB

1. **What is your educational level?**

A: No education experience B: Primary school and below

C: Junior middle school D: Senior high school E: University and up

1. **How long do you spend outdoor each day in the summer?**

A: Less than an hour B: 1–3 hours

C: 3–6 hours D: 6–8 hours E: More than 8 hours

1. **Do you have a chronic disease (e.g.,** **cardiovascular, respiratory) ？ YES/NO**
2. **What’s the building area of your house?**

A: less than 50 m^2^ B: 50–100 m^2^

C: 100–150 m^2^ D: 150–200 m^2^ E: more than 200 m^2^

1. **Please tick "√" next to the corresponding number**

|  | **Never** | **Seldom** | **General** | **More** | **Always** |
| --- | --- | --- | --- | --- | --- |
| 1. Frequency of obtaining hot weather information initiatively (For example, by mobile, TV, computer) | 1 | 2 | 3 | 4 | 5 |
| 2. Go out for cooling centers initiatively (For example, river-waterfront spaces, parks, markets, mountains) | 1 | 2 | 3 | 4 | 5 |
| 3. Frequency of hot weather information, education and warnings provided by your community groups | 1 | 2 | 3 | 4 | 5 |
| 4. Frequency of street sprinkling operations by municipal departments | 1 | 2 | 3 | 4 | 5 |
|  | **Very Hard** | **Hard** | **General** | **Easy** | **Very easy** |
| 5. Is it convenient for you to access cooling facilities? (Parks, markets) | 1 | 2 | 3 | 4 | 5 |
| 6. Is it convenient for you to access medical support facilities? | 1 | 2 | 3 | 4 | 5 |
| 7. Is it convenient for you to access public transportation facilities? | 1 | 2 | 3 | 4 | 5 |
| 8. Is it convenient for you to access river-waterfront spaces? | 1 | 2 | 3 | 4 | 5 |
| 9. Is it convenient for you to access green spaces? | 1 | 2 | 3 | 4 | 5 |

1. **Based on your previous answers, please make a comprehensive judgment on your adaptive capacity to hot weather in the summer?**

A: Lowest B: Low C: Medium D: High E: Highest

**Thank you!**
