# Supplementary material for: Heat Adaptive Capacity: What Causes the Differences Between Residents of Xiamen Island and Other Areas?
Source: Front Public Health. 2022 Feb 21;10:799365. doi: 10.3389/fpubh.2022.799365 (PMC8899036; doi:10.3389/fpubh.2022.799365)
Supplement: Supplementary file 2 [file Data_Sheet_2.docx]

Supplementary Material S2

# Supplementary Tables

**Table S2| Basic characteristics of participants (continuous variables).**

| **Characteristics** | ***n ^a^*** | **% *^b^*** | **Mean** | **Test Statistics, *p*-value** |
| --- | --- | --- | --- | --- |
| **Body Mass Index** |  |  |  | F = 0.770; 0.381 |
| Xiamen Island | 298 | 97.39% | 21.91 |  |
| Other Areas of Xiamen | 315 | 97.22% | 26.94 |  |
| **Number of family members** |  |  |  | F = 2.973; 0.085 |
| Xiamen Island | 297 | 97.06% | 3.39 |  |
| Other Areas of Xiamen | 312 | 96.40% | 3.26 |  |
| **Number of air conditioners in household** |  |  |  | F = 0.001; 0.987 |
| Xiamen Island | 290 | 94.77% | 2.43 |  |
| Other Areas of Xiamen | 307 | 94.75% | 2.43 |  |
| **Number of fans in household** |  |  |  | F = 7.334; < 0.01 |
| Xiamen Island | 294 | 96.08% | 1.88 |  |
| Other Areas of Xiamen | 308 | 95.06% | 1.58 |  |
| **Number of rooms in household** |  |  |  | F = 1.723; 0.190 |
| Xiamen Island | 295 | 96.40% | 3.07 |  |
| Other Areas of Xiamen | 312 | 96.30% | 2.79 |  |

^a^ Number of participants for each category; ^b^ Percentage of participants for each category; F = ANOVA. Remarks: Percentages may not add up to 100% due to missing data from a very small number of participants who did not fill in all the options.

**Table S3 |** **Main characteristics of participants (classified variables).**

| **Characteristics** | **Xiamen Island** | | **Other Areas of Xiamen** | | **Test Statistics, *p*‐Value** |
| --- | --- | --- | --- | --- | --- |
|  | ***n ^a^*** | **% *^b^*** | ***n ^a^*** | **% *^b^*** |  |
| **Gender** |  |  |  |  | χ^2^ = 0.059; 0.808 |
| Men | 168 | 54.90% | 181 | 55.86% |  |
| Female | 138 | 45.10% | 143 | 44.14% |  |
| **Age** |  |  |  |  | χ^2^ = 1.263; 0.868 |
| 18 and Under 18 | 22 | 7.19% | 23 | 7.10% |  |
| 19–34 | 188 | 61.44% | 201 | 62.04% |  |
| 35–49 | 69 | 22.55% | 68 | 20.99% |  |
| 50–64 | 15 | 4.90% | 22 | 6.79% |  |
| 65 and Up 65 | 11 | 3.59% | 10 | 3.09% |  |
| **Education level** |  |  |  |  | χ^2^ = 3.413; 0.491 |
| No education experience | 1 | 0.33% | 3 | 0.93% |  |
| Primary school and below | 11 | 3.59% | 7 | 2.16% |  |
| Junior middle school | 37 | 12.09% | 50 | 15.43% |  |
| Senior high school | 74 | 24.18% | 76 | 23.46% |  |
| University and up | 182 | 59.48% | 187 | 57.72% |  |
| **Health status** |  |  |  |  | χ^2^ = 0.433; 0.510 |
| Yes | 29 | 9.48% | 26 | 8.02% |  |
| No | 276 | 90.20% | 298 | 91.98% |  |
| **Hours spent outdoors per day** |  |  |  |  | χ^2^ = 2.361; 0.670 |
| Less than an hour | 41 | 13.40% | 46 | 14.20% |  |
| 1–3 hours | 139 | 45.42% | 134 | 41.36% |  |
| 3–6 hours | 76 | 24.84% | 77 | 23.77% |  |
| 6–8 hours | 26 | 8.50% | 34 | 10.49% |  |
| More than 8 hours | 24 | 7.84% | 33 | 10.19% |  |
| **Monthly household income** |  |  |  |  | χ^2^ = 2.640; 0.620 |
| Less than RMB 2,000 | 27 | 8.82% | 27 | 8.33% |  |
| RMB 2,000–5,000 | 45 | 14.71% | 63 | 19.44% |  |
| RMB 5,000–10,000 | 99 | 32.35% | 107 | 33.02% |  |
| RMB 10,000–20,000 | 86 | 28.10% | 81 | 25.00% |  |
| More than RMB 20,000 | 37 | 12.09% | 40 | 12.35% |  |
| **Building area** |  |  |  |  | χ^2^ = 6.587; 0.159 |
| less than 50 m^2^ | 64 | 20.92% | 95 | 29.32% |  |
| 50–100 m^2^ | 109 | 35.62% | 114 | 35.19% |  |
| 100–150 m^2^ | 89 | 29.08% | 78 | 24.07% |  |
| 150–200 m^2^ | 23 | 7.52% | 20 | 6.17% |  |
| more than 200 m^2^ | 16 | 5.23% | 14 | 4.32% |  |
| **Years of local residence** |  |  |  |  | χ^2^ = 5.401; 0.249 |
| Less than a year | 31 | 10.13% | 46 | 14.20% |  |
| 1–3 years | 44 | 14.38% | 56 | 17.28% |  |
| 3–5 years | 43 | 14.05% | 50 | 15.43% |  |
| 5–10 years | 54 | 17.65% | 54 | 16.67% |  |
| More than 10 years | 133 | 43.46% | 117 | 36.11% |  |
| **Obtain heat information initiatively** |  |  |  |  | χ^2^ = 0.881; 0.927 |
| Never | 34 | 11.11% | 40 | 12.35% |  |
| Seldom | 91 | 29.74% | 102 | 31.48% |  |
| Fewer | 58 | 18.95% | 54 | 16.67% |  |
| More | 85 | 27.78% | 87 | 26.85% |  |
| Always | 37 | 12.09% | 40 | 12.35% |  |
| **Go out for cooling centers** |  |  |  |  | χ^2^ = 2.798; 0.592 |
| Never | 21 | 6.86% | 24 | 7.41% |  |
| Seldom | 101 | 33.01% | 116 | 35.80% |  |
| Fewer | 107 | 34.97% | 94 | 29.01% |  |
| More | 54 | 17.65% | 66 | 20.37% |  |
| Always | 20 | 6.54% | 22 | 6.79% |  |
| **Convenience of accessing cooling facilities** |  |  |  |  | χ^2^ = 1.149; 0.886 |
| Very hard | 11 | 3.59% | 14 | 4.32% |  |
| Hard | 40 | 13.07% | 36 | 11.11% |  |
| General | 52 | 16.99% | 50 | 15.43% |  |
| Easy | 146 | 47.71% | 158 | 48.77% |  |
| Very easy | 57 | 18.63% | 65 | 20.06% |  |
| **Convenience of accessing medical support facilities** |  |  |  |  | χ^2^ = 2.347; 0.672 |
| Very hard | 12 | 3.92% | 9 | 2.78% |  |
| Hard | 38 | 12.42% | 34 | 10.49% |  |
| General | 49 | 16.01% | 60 | 18.52% |  |
| Easy | 149 | 48.69% | 168 | 51.85% |  |
| Very easy | 54 | 17.65% | 51 | 15.74% |  |
| **Convenience of accessing public transportation facilities** |  |  |  |  | χ^2^ = 7.907; 0.095 |
| Very hard | 5 | 1.63% | 16 | 4.94% |  |
| Hard | 19 | 6.21% | 13 | 4.01% |  |
| General | 31 | 10.13% | 40 | 12.35% |  |
| Easy | 160 | 52.29% | 169 | 52.16% |  |
| Very easy | 90 | 29.41% | 85 | 26.23% |  |
| **Convenience of accessing river-waterfront spaces** |  |  |  |  | χ^2^ = 1.988; 0.738 |
| Very hard | 19 | 6.21% | 28 | 8.64% |  |
| Hard | 52 | 16.99% | 59 | 18.21% |  |
| General | 61 | 19.93% | 56 | 17.28% |  |
| Easy | 119 | 38.89% | 125 | 38.58% |  |
| Very easy | 52 | 16.99% | 54 | 16.67% |  |
| **Convenience of accessing green spaces** |  |  |  |  | χ^2^ = 9.502; 0.050 |
| Very hard | 7 | 2.29% | 18 | 5.56% |  |
| Hard | 40 | 13.07% | 26 | 8.02% |  |
| General | 50 | 16.34% | 44 | 13.58% |  |
| Easy | 143 | 46.73% | 159 | 49.07% |  |
| Very easy | 64 | 20.92% | 76 | 23.46% |  |
| **Frequency of releasing hot weather information initiatively** |  |  |  |  | χ^2^ = 1.015; 0.908 |
| Never | 47 | 15.36% | 57 | 17.59% |  |
| Seldom | 103 | 33.66% | 103 | 31.79% |  |
| Fewer | 95 | 31.05% | 95 | 29.32% |  |
| More | 48 | 15.69% | 54 | 16.67% |  |
| Always | 8 | 2.61% | 10 | 3.09% |  |
| **Frequency of street sprinkling operations by municipal departments** |  |  |  |  | χ^2^ = 1.659; 0.798 |
| Never | 25 | 8.17% | 27 | 8.33% |  |
| Seldom | 82 | 26.80% | 81 | 25.00% |  |
| Fewer | 71 | 23.20% | 78 | 24.07% |  |
| More | 91 | 29.74% | 108 | 33.33% |  |
| Always | 31 | 10.13% | 26 | 8.02% |  |
| **Heat Adaptive Capacity** |  |  |  |  | χ^2^ = 3.930; 0.415 |
| Lowest | 5 | 1.63% | 4 | 1.23% |  |
| Low | 15 | 4.90% | 26 | 8.02% |  |
| Medium | 144 | 47.06% | 159 | 49.07% |  |
| High | 122 | 39.97% | 112 | 34.57% |  |
| Highest | 20 | 6.54% | 23 | 7.10% |  |

^a^ Number of participants for each category; ^b^ Percentage of participants for each category; χ^2^ = Chi-square test. Remarks: Percentages may not add up to 100% due to missing data from a very small number of participants who did not fill in all the options.
